# HPV Population Profiling in Healthy Men by Next-Generation Deep Sequencing Coupled with HPV-QUEST

**DOI:** 10.3390/v8020028

**Published:** 2016-01-25

**Authors:** Li Yin, Jin Yao, Kaifen Chang, Brent P. Gardner, Fahong Yu, Anna R. Giuliano, Maureen M. Goodenow

**Affiliations:** 1 Department of Pathology, Immunology & Laboratory Medicine and UF Health Cancer Center, University of Florida, Gainesville, FL 32610, USA; kaifen@pathology.ufl.edu (K.C.); brentga@pcom.edu (B.P.G.); goodenow@ufl.edu (M.M.G.); 2 Interdisciplinary Center for Biotechnology Research, University of Florida, Gainesville, FL 32610, USA; jin.yao2009@gmail.com (J.Y.); fyu@ufl.edu (F.Y.); 3 Center for Infection Research in Cancer at H. Lee Moffitt Cancer Center, Tampa, FL 33612, USA; Anna.Giuliano@moffitt.org

**Keywords:** HPV, multiple-type HPV infection, asymptomatic men, deep sequencing, HPV-QUEST, Linear Array, Papillomavirus Episteme

## Abstract

Multiple-type human papillomaviruses (HPV) infection presents a greater risk for persistence in asymptomatic individuals and may accelerate cancer development. To extend the scope of HPV types defined by probe-based assays, multiplexing deep sequencing of HPV L1, coupled with an HPV-QUEST genotyping server and a bioinformatic pipeline, was established and applied to survey the diversity of HPV genotypes among a subset of healthy men from the HPV in Men (HIM) Multinational Study. Twenty-one HPV genotypes (12 high-risk and 9 low-risk) were detected in the genital area from 18 asymptomatic individuals. A single HPV type, either HPV16, HPV6b or HPV83, was detected in 7 individuals, while coinfection by 2 to 5 high-risk and/or low-risk genotypes was identified in the other 11 participants. In two individuals studied for over one year, HPV16 persisted, while fluctuations of coinfecting genotypes occurred. HPV L1 regions were generally identical between query and reference sequences, although nonsynonymous and synonymous nucleotide polymorphisms of HPV16, 18, 31, 35h, 59, 70, 73, cand85, 6b, 62, 81, 83, cand89 or JEB2 L1 genotypes, mostly unidentified by linear array, were evident. Deep sequencing coupled with HPV-QUEST provides efficient and unambiguous classification of HPV genotypes in multiple-type HPV infection in host ecosystems.

## 1. Introduction

Human papillomaviruses (HPV) are globally ubiquitous DNA viruses found in mucosal and cutaneous anatomical sites and comprised by more than a hundred genotypes. Low-risk HPV genotypes are associated with genital warts, while high-risk HPV genotypes, in particular HPV16, cause a number of cancers in men and women [1,2,3,4,5,6,7]. Coinfection by multiple-type high-risk HPV, alone or in combination with low-risk HPV, is associated with precancerous lesions, accelerates development of cancers [8,9,10,11,12], and complicates treatment outcomes [4,13,14,15,16,17,18]. Multiple-type high-risk and/or low-risk HPV infection also occurs in asymptomatic individuals and is associated with a greater risk of persistent infection [19,20] that correlates with cancer development [21,22,23,24,25]. Consequently, identifying infection as multiple HPV types prior to development of symptoms has explicit clinical value. 

Studies of multiple-type HPV infection by conventional probe-based assays are hampered by the limited scope of type-specific probes and/or cross-hybridization by probes for related HPV genomes [26,27,28,29,30]. Direct sequencing based on electropherographs or pyrograms is problematic in accurately defining multiple infections present in one specimen [31,32]. Next-generation deep sequencing (NGS) technology combines the specificity of sequence data with a capacity to analyze large numbers of samples within a clinical setting. In this study, an HPV-genotyping system was developed by coupling NGS with HPV-QUEST, a web server we developed previously for automated HPV genotyping with a capability of handling up to 10 megabases (Mb) of sequences [33], and applied to a subset of healthy men from the HPV in Men (HIM) Multinational Study [34,35,36,37] to survey HPV genotype diversity cross-sectionally and longitudinally.

## 2. Materials and Methods

### 2.1. Study Participants, Specimen Collection, and DNA Extraction

The study included 22 samples from 18 men from the United States (n = 15), Brazil (n = 1), or Mexico (n = 2), representing a subset of healthy males from the HPV in Men (HIM) Multinational Study recruited between 2005 and 2008 to examine the HPV prevalence in asymptomatic males [34,35,36,37]. The HIM study was approved by the human-subjects’ committees of the University of South Florida (St. Petersburg, FL, USA), the Ludwig Institute for Cancer Research (São Paulo, Brazil), the Centro de Referẽncia e Tratamento de Doencas Sexualmente Transmissiveis e AIDS, and the National Institute of Public Health of Mexico (Cuernavaca, Mexico). Written consent was obtained from all participants. Participants at study entry were a median (25%–75% quartile range (QR)) age of 22 years (19–31 years), had none to 3 sexual partners within 6 weeks before sampling (Table S1), were HIV-1 negative, and received no HPV vaccines before or during the course of the study. Genital sampling and DNA extraction have been described previously [35,38]. In brief, saline-wetted Dacron applicators (Digene, Gaithersburg, MD, USA) were used to swab three sites of the external genitalia (glans penis/coronal sulcus, penile shaft, and scrotum), and combined into one sample in 450 µL of specimen transport medium (Digene Corporation, Gaithersburg, MD). DNA was extracted using the Media Kit (Qiagen, Valencia, CA, USA) according to the manufacturer’s instructions, and eluted in a 100- to 120 μL elution buffer. The consistency of sample collection reached 91.4% as tested previously [38]. To prevent sample cross-contamination, DNA extraction was carefully performed in a clean and plasmid-free environment. HPV L1 genotyping by linear array (LA) method (Roche Diagnostics, Indianapolis, IN, USA) using PGMY11/09 (PGMY) consensus primers and probes for 37 HPV mucosal types (6, 11, 16, 18, 26, 31, 33, 35, 39, 40, 42, 45, 51–56, 58, 59, 61, 62, 64, 66–72, 73 (MM9), 81, 82 (MM4), 83 (MM7), 84 (MM8), IS39 and CP6108) (Linear Array® HPV Genotyping Test, Roche Molecular Diagnostics, Pleasanton, CA, USA) was performed at the H. Lee Moffitt Cancer Center (HLMCC, Tampa, FL, USA). HPV genotyping by LA was highly reproducible with 83.5% or 93.2% concordance rate for single- or multiple-type detection, respectively [38]. The deep sequencing sub-study included samples with HPV L1 genomes identified by the LA method (12 samples including longitudinal samples from 8 participants) or with indeterminate HPV infection (10 samples from 10 participants) reflecting failure either to amplify by PGMY primers or to hybridize by probes in the LA assay. DNA from de-identified samples, provided to the University of Florida (UF) with approval by the Institutional Review Boards at HLMCC and UF, was concentrated by speed vacuum, re-suspended in 5 µL of double distilled water, and used in a single PCR to capture all HPV copies in the sample. The median (QR) input of total DNA was 349 ng (182–995 ng), equivalent to approximately median (QR) of 72,000 cells (31,500–150,000 cells), based on a calculation that 600 ng DNA is equivalent to 100,000 cells [39,40,41,42]. Limited sample DNA precluded direct quantification of total cells or HPV genome copies by quantitative PCR. Deep sequencing coupled with HPV-QUEST [33] was applied to a cross-sectional study of 18 individuals, and a longitudinal follow-up of 2 participants with 6 samples obtained at 6-month intervals. DNA extracted from epidermoid cervical cancer cell lines CaSki or C-4 II (ATCC, Manassas, VA, USA) was included in the study for sequence pipeline development and genotyping verification [43].

### 2.2. Generation of HPV L1 Amplicon Libraries and Deep Sequencing

**Figure 1:**
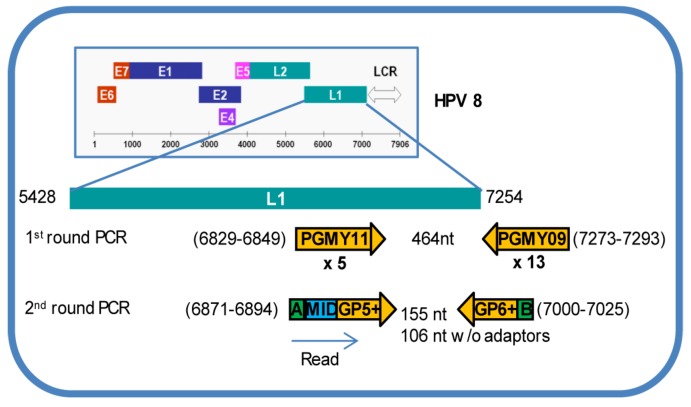
Primer sets used to generate human papillomaviruses (HPV) L1 amplicon libraries. A cocktail of PGMY11/09 PGMY consensus primers was used in 1st-round amplification to produce a product of 464 nucleotides (nt). GP5+ and GP6+ consensus primers conjugated at 5’ end with adaptors A or B respectively, were used in 2nd-round amplification to produce a product of 155 nt (106 nt w/o adaptors, multiplex identifier (MID) and primers). Primer positions are based on HPV8 genome (M12737.1) [44,45]. Distinct MIDs were added between adaptor A and GP5+ for sample identification. Sequences were read from adaptor A.

Nested PCR with PGMY [44] followed by GP5+/6+ (GP+) [45] was used to generate HPV L1 amplicon libraries because PGMY/GP+ has greater sensitivity and broader coverage of HPV genotypes compared to PGMY alone, GP+ alone, MY11/09 (MY) alone or MY/GP+ [46,47]. In addition, PGMY/GP+ amplicon length of 150 nucleotides (nt) falls within the average read length of 400 nt by Titanium pyrosequencing. First-round amplification applied Expand High Fidelity PCR System (Roche, Branchburg, NJ, USA). Ten percent of the product was then used for 2nd-round amplification. Sensitivity of HPV DNA detection reached a single copy based on an amplification of a serially diluted HPV16 L1 clone constructed from PGMY-amplified CaSki DNA by TOPO TA cloning (Invitrogen, Grand Island, NY, USA), which is in agreement with data published previously [46]. Inter- and intra-library agreements were over 90%, as evaluated previously [48,49], which insures reproducibility of HPV testing by deep sequencing. For Titanium amplicon deep sequencing, 5’ ends of PG5+ and PG6+ primers were conjugated with 25-mer nucleotide adaptors A (CGTATCGCCTCCCTCGCGCCATCAG) or B (CTATGCGCCTTGCCAGCCCGCTCAG), respectively. Multiplex identifier (MID) ten-mer nucleotides were added between adaptor A and the GP5+ sequence to allow for automated software identification of samples after pooling for multiplex deep sequencing [50]. Primer pairs produced HPV L1 amplicons of 106 nt excluding adaptors, MID and primers (Figure 1). First-round PCR included denaturation at 95 °C for 9 min, followed by 40 cycles of 95 °C for 30 s, 55 °C for 1 min, and 68 °C for 1 min, and a final extension at 68 °C for 5 min. Second-round PCR included denaturation at 95 °C for 2 min, followed by 40 cycles of 95 °C for 1 min, 56 °C for 1 min and 68 °C for 1 min, with a final extension at 68 °C for 10 min. Although 40 °C annealing temperature for GP5+/6+ PCR amplifications was established in 1995 [45], our analysis, using Oligo 5.0 (Molecular Biology Insights, Inc., Colorado Springs, CO, USA) of GP5+/6+ annealing efficiencies for all 37 HPV genotypes included in the LA probe set, defined the optimal median (QR) annealing temperature as 47 °C (46–48 °C) and the maximum median (QR) annealing temperature as 62 °C (60–65 °C). To increase PCR efficiency, reduce nonspecific amplification, and accommodate annealing between the HPV L1 region, the 10-mer barcode and the 25-mer adaptor incorporated into the primers, an annealing temperature of 56 °C, between optimal and maximum for all HPV types tested, was used. This annealing temperature was able to detect HPV56, which has the lowest optimal annealing temperature (44 °C) among the 37 HPV types included in LA probe set. HPV L1 DNA amplicon libraries were generated successfully in cross-sectional samples from 18 participants, 6 longitudinal samples from 2 individuals, cell lines CaSki and C-4 II, and an HPV16 L1 clone (Table S1).

Standard operating procedures were followed at every step of PCR amplifications to prevent cross-contamination, and no template controls were always negative.

Gel-purified amplicons were sequenced from adaptor A using a Genome Sequencer FLX (454 Life Sciences, Branford, CT, USA) with Titanium chemistry (Roche) according to the manufacturer’s protocol in the University of Florida Interdisciplinary Center for Biotechnology Research (ICBR, Gainesville, FL, USA). Multiplex deep sequencing was performed on HPV L1 libraries pooled at equal molar ratio at the bead recovery step, a critical modification as pooling samples at the DNA level produced skewed sequencing output (data not shown). The first 14 samples were pooled in one region of an 8-region PicoTiter plate to obtain a median (QR) of 1,562 (792–1,831) raw reads per sample. To increase the sequence depth and accelerate the turnaround time in the ICBR Sequencing Core Facility, the second set of 11 samples, including 8 samples from 4 participants, 2 control cell lines, and 1 HPV16 molecular clone were sequenced in two regions of a 4-region PicoTiter plate to generate a median (QR) of 26,193 (16,586–36,771) raw reads per sample (Table S1). All raw reads obtained had been prefiltered based on Phred Quality Scores to exclude any reads with >50% of base calls with Phred quality scores less than 40.

### 2.3. Sequence Analysis

**Figure 2:**
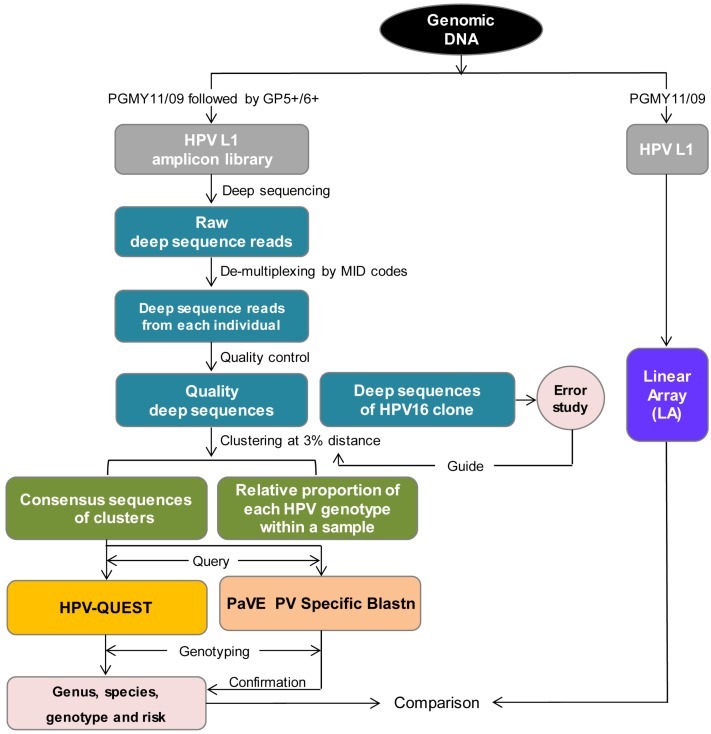
Bioinformatic pipeline of sequence analysis. HPV L1 was amplified by PCR using PGMY or nested PCR using PGMY/GP+ from genomic DNA from each individual and subjected to Linear Array (LA) and deep sequencing/HPV-QUEST, respectively, to compare sensitivity and coverage in HPV genotype capturing. Raw deep sequencing reads generated from pooled HPV L1 libraries were separated and assigned to each individual according to MID sequence codes. Quality control removed low quality reads. Quality sequences of an HPV16 clone were aligned to HPV16 reference sequence (GI│333031) to study errors due to PCR and deep sequencing. To study HPV L1 sequences at variant level, quality sequences were clustered at 3% pairwise distance [51]. Consensus sequence derived from each cluster represented an HPV variant, and relative proportion of each HPV genotype within a sample served as a reference rather than a quantification of viral population structure. Consensus sequences were queried in HPV-QUEST to obtain HPV genus, species, genotype and oncogenicity. Papillomavirus Episteme (PaVE) Specific Blastn was used to confirm HPV-QUEST genotyping results, and Nationa Center for Biotechnology Information (NCBI) Blastn was applied to evaluate discrepancies of genotyping by HPV-QUEST and PaVE. NGS/HPV-QUEST was compared with LA with respect of sensitivity and coverage in identifying HPV genotypes and variants.

The bioinformatic pipeline for analysis of L1 deep sequences is outlined in Figure 2. Raw reads generated from pooled libraries were de-multiplexed in Geneious Pro 5.6.5 (Biomatters Ltd., Auckland, New Zealand) using “Separate Reads by Barcode” function with 454 MIDS selected as the Barcode Set to assign sequences to each individual. Quality control steps were performed first in Geneious Pro 5.6.5 to exclude unassigned reads, reads inaccurately assigned to MIDs unused in multiplexing due to an incomplete MID, no MID, sequencing errors in the MID, reads with more than one mismatch in any of the primers, or reads with a sequence length outside the mean ± 2 SD range. Subsequent analysis in BioEdit (Ibis Biosciences, Carlsbad, CA, USA) identified and excluded reads with ambiguous nucleotide(s) or out-of-reading-frame indels. After quality control, a median (QR) of 1,441 (684–1,670) and 25,928 (16,444–36,038) quality sequences were obtained for the first 14 samples and the 2nd 11 samples, respectively, with correspondent median (QR) removal rate of 9.7% (8.0%–14.5%) and 1.4% (0.7%–3.4%) (Table S1).

To assess deep sequencing errors, 25,928 quality L1 deep sequences from a molecular clone of HPV16 were aligned to an HPV16 reference sequence (GI│333031) using MUSCLE in Geneious package, followed by BioEdit (Ibis Biosciences, Carlsbad, CA, USA). Errors were evaluated using an in-house code. Overall sequencing error rate was 0.46% (46 errors/10,000 nucleotides) with 0.09%, 0.03%, 0.08% and 0.26% for transitions, transversions, and insertions or deletions (indels), respectively. After correction of indels, rate of misincorporation was 0.12% (12 errors per 10,000 nucleotides), which is 18 times lower than 2%, the maximum dissimilarity within an HPV genotype [52,53,54].

Quality sequences were clustered at 3% pairwise distance (representing 2% dissimilarity within variants [52,53,54] plus 1% to more than compensate for sequencing errors) using ESPRIT [51]. ESPRIT generated a consensus sequence for each cluster representing an HPV genotype variant, and displayed the size of the cluster (number of sequences/cluster) reflecting the abundance of each variant, which only serves as a reference rather than a quantitation due to the possible bias of PGMY/GP+ consensus primers towards certain HPV genotypes over others. The number of consensus sequences for each genotype is detailed in Table S1.

Consensus sequences were queried in HPV-QUEST, a custom HPV-genotyping server which collected 150 cutaneous and mucosal HPV reference sequences containing the L1 region obtained from National Center for Biotechnology Information (NCBI) Genebank [55], Los Alamos HPV Sequence Database [56] and Virus Sequence Database [57] with nomenclatures updated, and accommodates large sequence data sets for automated and expedited HPV genotyping and classification of HPV genus, species, type and oncogenicity (high risk = oncogenic; low risk = nononcogenic) [33]. To insure reliability of the HPV genotyping, highly stringent cutoff values were applied with e-value ≤ 1.0E-38 and ≥ 80 nucleotide identities between query (~106 nt) and reference sequences. Because of stability and conservation of HPV genomes over evolutionary times [58], genome segments as short as 20 to 30 nt can provide reliable genotyping [26,31]. Validation analysis of genetically unrelated sequences, including human immunoglobulin heavy chain variable region or human immunodeficiency virus type 1 envelope hypervariable region 3, returned results as “not identified (ND)”. Singleton HPV genotypes concordant with LA typing, or unique among individuals, or containing sample-specific substitution(s) were included in the study because of the unlikelihood of contamination among the samples. They were otherwise excluded from further analysis. Cervical epidermoid carcinoma cell lines CaSki and C-4 II, reported to contain HPV16 or HPV18, respectively, were used to verify the sequence analysis pipeline. A total of 16,444 quality sequences generated from C-4 II DNA were genotyped as a single variant identical to reference HPV18, while a total of 22,763 quality sequences obtained from CaSki DNA formed a single cluster that was identical to reference HPV16 (Tables S1 and S2). Papillomavirus Episteme **(**PaVE) PV Specific Blastn [59] was used to confirm HPV-QUEST genotyping results, and NCBI Blastn [60] was applied to evaluate discrepancies of genotyping by HPV-QUEST and PaVE PV Specific Blastn. Genotype variants were studied by aligning them to correspondent reference sequences to identify synonymous and/or nonsynonymous substitution(s).

## 3. Results

### 3.1. HPV Genotype Infection and Diversity

**Figure 3:**
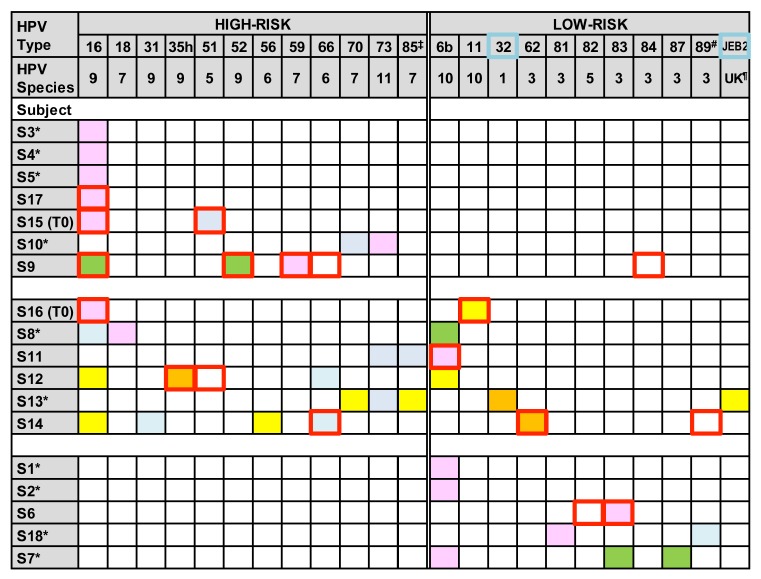
HPV genotyping by deep sequencing/HPV-QUEST. Each HPV genotype captured by deep sequencing within an individual is depicted as a color-filled block: Pink represents HPV type defined by ≥95% of sequences; orange, sequences ≥50% but <95%; yellow, sequences ≥1% but <50%; green, sequences ≥0.1% but <1%; and blue, <0.1% sequences. Red-outlined squares represent HPV types detected by LA. All HPV types, except for HPV32 and JEB2 outlined by blue, are covered by LA probe set. Symbols: *, sample untyped by LA; ‡, HPVcand85; #, HPVcand89; ¶, unknown. Reference sequences for genotypes identified in the study samples include: HPV16 (GI|333031), HPV18 (GI|60975), HPV31 (GI|333048), HPV35h (GI|396997), HPV51 (GI|333087), HPV52 (GI|397038), HPV56 (GI|39053), HPV59 (GI|557236), HPV66 (GI|1020290), HPV70 (GI|1173493), HPV73 (GI|1491692), HPVcand85 (GI|4574720), HPV6b (GI|60955), HPV11 (GI|333026), HPV32 (GI|396981), HPV62 (GI|577400), HPV81 (GI|40804509), HPV83 (GI|5059324), HPV87 (GI|14475578), HPVcand89 (GI|22095322), or JEB2 (GI|45925861) [53,61,62,63].

HPV genotype infection and diversity were investigated in cross-section DNA samples from 18 asymptomatic participants. A total of 21 (12 high-risk and 9 low-risk) HPV genotypes, representing 9 species (1, 3, 5, 6, 7, 9, 10, 11 or unknown) were detected (Figure 3). Seven individuals were infected by a single HPV type, either HPV16 (S3–S5 and S17), HPV6b (S1 and S2), or HPV83 (S6). In contrast, infection by multiple different HPV genotypes was detected in 11 of 18 individuals. Four individuals (S10, S15 (T0), S16 (T0) and S18) had two HPV genotypes, four individuals (S7–S9 and S11) had 3 genotypes, one (S12) had 4 genotypes, and two (S13 and S14) had 5 genotypes. The diversity of HPV populations in the samples was independent of the number of deep sequences evaluated, number of sexual partners, or participant age (Table S1). Overall, NGS/HPV-QUEST identified HPV genotypes in all individuals, including a number of genotypes undetermined by LA.

A majority (12 of 18, 67%) of the cohort had exclusively high- or low-risk genotypes, including seven individuals (S3–S5, S9, S10, S15 (T0) and S17) with high-risk HPVs16, 51, 52, 59, 70 and 73 genotypes, and five (S1, S2, S6, S7 and S18) with low-risk HPVs6b, 81, 83, 87 or cand89 genotypes (Figure 3). In contrast, 33% (6 of 18) of individuals (S8, S11–S14 and S16 (T0)) had combinations of high- and low-risk genotypes, including three individuals (S12, S13 and S14) with as many as 3 to 4 high-risk genotypes in combination with 1 or 2 low-risk genotypes. Vaccine high-risk HPV16 or HPV18 were identified in 10 of 13 individuals with high-risk types, while low-risk vaccine types, HPV6b or HPV11, appeared in 7 of 11 individuals with low-risk types. 

Results of the NGS/HPV-QUEST typing were concordant with LA genotyping for 10 high- or low-risk genotypes (HPVs16, 35h, 51, 52, 59, 66, 6b, 11, 62 and 83) in 8 individuals (S6, S9, S11, S12, and S14–S17) (Figure 3). While two low-risk HPVs82 and 84 were classified by LA but not by deep sequencing, six high-risk (HPVs18, 31, 56, 70, 73 and cand85) and two low-risk genotypes (HPVs81 and 87) that were unclassified by LA were identified by deep sequencing. In addition, HPV32 and HPVJEB2, not included among the LA probes, were identified by deep sequencing.

Genotypes defined by PaVE PV Specific Blastn were virtually identical to those classified by HPV-QUEST with exactly the same local identities to the same reference sequences used in both databases. Minor differences between the two genotyping tools, including HPVJEB2 typed by HPV-QUEST and classified as HPV72 by PaVE PV Specific Blastn, were due to different reference sequences used by the two programs (Table S2). HPVJEB2 is more accurate because the local identity is higher and is the top hit by NCBI Blastn.

### 3.2. HPV Genotype Variants

**Figure 4:**
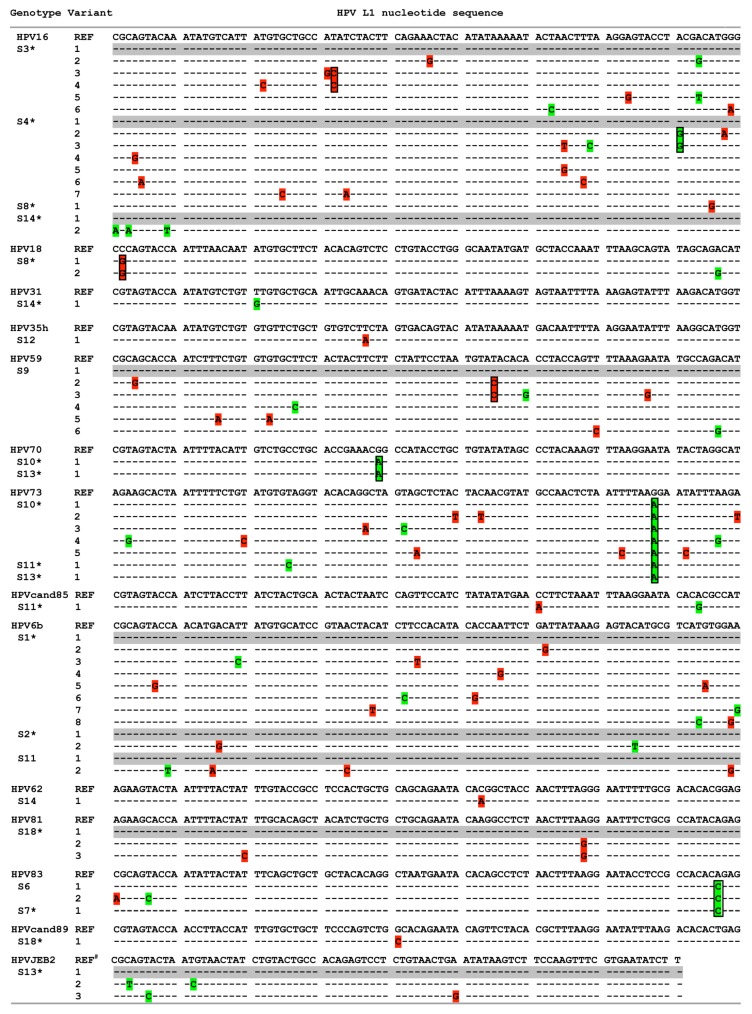
HPV L1 variants within genotypes. HPV L1 nucleotide sequence(s) of genotype variant(s) were aligned to corresponding reference genotype sequences with grey color highlighting variants identical to reference sequences. Synonymous and nonsynonymous nucleotide differences were highlighted by green and red, respectively, and boxed to indicate the same nucleotide substitution in more than one variant of an HPV genotype in one or multiple individuals. Nucleotide positions in HPV L1 sequences were based on L1 region of reference sequences (nucleotides 1123 to 1212 for HPV16; 1195 to 1290, HPV18; 1051 to 1137, HPV31; 1006 to 1098, HPV35h; 1052 to 1141, HPV59; 1006 to 1095, HPV70; 1006 to 1095, HPV73; 1012 to 1098 for HPVcand85; 1000 to 1089 for HPV6b; 67 to 156 for HPV62; 1018 to 1107 for HPV81; 1018 to 1107 for HPV 83; 1066 to 1153 for HPVcand89, and 15 to 95 for HPVJEB2 (#: partial L1 reference sequence)). * sample with variant(s) failed to be typed by LA.

Across the study group, L1 regions from a variety of genotypes were identical to reference sequences at the nucleotide level (for example, HPVs51, 52, 56, 66, 11, 32 and 87 from S7, S9 and S12–S16; HPV16 from S5, S9, S12 and S15–S17; HPVcand85 from S13; and HPV6b from S7, S8 and S12). In contrast, a number of L1 variants displayed nucleotide differences from the corresponding reference sequences in more than 77% (14 of 18) of individuals (Figure 4). Both synonymous and nonsynonymous nucleotide variants were detected within high-risk and low-risk HPV genotypes. Multiple sample-specific substitutions could be found within a single HPV genotype, such as HPV16 in S3 (T1154C) and S4 (A1203G), HPV18 in S8 (C1196G), HPV59 in S9 (T1115C), and HPV81 in S18 (A1085G), while same substitution within a genotype occurred in samples from different individuals, such as HPV70 in S10 and S13 (G1044A), HPV73 in S10, S11 and S13 (G1083A), or HPV83 in S6 and S7 (A1104C). Overall, 81% (47/58) of nucleotide variants differing from reference variants within HPV L1 were identified among samples unclassified by LA. 

### 3.3. Longitudinal Changes of HPV Types within Two Healthy Individuals

**Figure 5:**
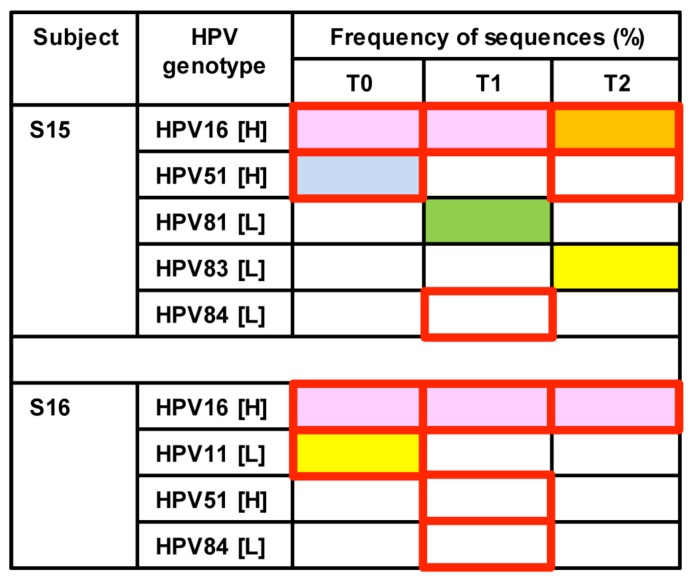
Longitudinal changes of HPV genotypes in two individuals. Each HPV genotype captured by deep sequencing within an individual is depicted as a color-filled block: Pink represents HPV type defined by ≥95% of sequences; orange, sequences ≥50% but <95%; yellow, sequences ≥1% but <50%; green, sequences ≥0.1% but <1%; and blue, <0.1% sequences. Red-outlined squares represent HPV types detected by LA. H: high-risk; L: low-risk.

Longitudinal changes of HPV genotypes were evaluated in S15 and S16 based on the ability to generate amplicon libraries from a series of three samples collected at six-month intervals (T0, T1, and T2) (Figure 5). Similar to cross-sectional results, HPV84 genotype captured by LA was not detected by deep sequencing in either participant. In contrast, HPV16 appeared as the persistent genotype at each time point with temporal fluctuations in other genotypes, including HPVs51, 81, and 83 in S15 or HPV11 in S16. All HPV genotypes identified in the samples over time from each individual displayed nucleotide identities with reference genotypes. 

## 4. Discussion

Much recent attention has highlighted the necessity of HPV genotyping in clinical pathology. An increasing line of evidence implicates HPV viral diversity and genotypes as substantial factors in cancer grade, response to therapy, recurrence, and a patient’s survival in HPV-related cancers, e.g., cervical cancers (CC) and head and neck squamous cell carcinomas (HNSCC) [12,13,14,15,16,17,18,30,64]. Infection by multiple HPV genotypes leads to poor response to radiotherapy with reduced survival in CC [13,14], and α7 genotypes, e.g., HPVs18, 39 and 45 are more resistant to radiotherapy [14,30]. HPV^+^ HNSCC are emerging as a separate biological entity among all HNSCC. HPV-positive HNSCC are less likely to harbor p53 mutations, are more sensitive to radio/chemotherapy, and have a lower risk of recurrence and a better chance of survival than HPV-negative HNSCC [4,15,16,17]. Presence of low-risk HPV type(s) in HNSCC result in poor therapy outcome [18]. The College of American Pathologists has recently recommended routine HPV testing as part of the standard pathologic evaluation of resected oropharyngeal SCC [65]. 

HPV-genotyping methods vary considerably across laboratories, resulting in substantial disagreement among studies [65,66,67]. Hybridization- and sequence-based tests are two major classes of HPV-genotyping systems. Current conventional hybridization-based HPV-genotyping methods represented by LA (Roche), SPF_10_-LiPA_25_ [68,69], HPV DNA chip [70], and GP+ reverse line blot [71] detect a limited number of 22 to 37 HPV types out of more than 100 known mucosal types due to a limited number of type-specific probes available. Cross-hybridization between related HPV genomes compromises genotyping accuracy [37,64,72,73]. Primers and probes differ with PGMY11/09 for LA, SPF_10_ for SPF_10_-LiPA_25_, and GP+ for HPV DNA chip and GP+ reverse line blot, resulting in large differences in sensitivity of detecting HPV infection and individual genotypes among the tests [14,27,28]. 

With the rapid revolution in technology, NGS applied to HPV genotyping provides high sensitivity in capturing even low frequency types, reflecting unprecedented coverage of viral copies and almost no limit in the number of types detected in a sample by querying reference sequence databases [72,74,75,76]. NGS has opened up the opportunity to examine viral diversity, monitor persistence, fluctuation and evolution, discover new genotypes and survey vaccine response, which can be detailed at variant level [74,75,77,78]. Studies at the HPV variant level have clinical relevance since some HPV intra-type variants are geographically specific, associated with oncogenicity, or responsible for vaccine escape [52,79,80,81,82]. However, the spectrum of HPV genotypes identified by deep sequencing still relies on the primer system used for amplicon generation. 

In this study, we applied nested PCR using PGMY, followed by GP+ primers, to generate HPV L1 amplicon libraries since PGMY/GP+ provided greater sensitivity and broader coverage of HPV genotypes compared to MY alone, PGMY alone, GP+ alone, or MY/GP+ [46,47]. Other pyrosequencing-based HPV-genotyping studies used single-round amplification with MY, PGMY, or GP+ primers [72,74,76,83]. In addition, a 150 nt amplicon length falls within the average read length of 400 nt by Titanium pyrosequencing. Consensus primer systems all suffer from certain amplification bias towards HPV genotypes with greater priming efficiency [44,46,47,72,76,84,85]; thus, the percent of sequences may not correlate with the actual amount of virus present and cannot serve as a quantitative measure of viral population structure [72]. Primer design remains a critical factor for HPV genotyping by deep sequencing. Application of rolling circle amplification using random hexamer followed by deep sequencing may provide unbiased amplification of episomal HPV particles but misses genotypes integrated into the cell genome, which are closely correlated with viral persistence, cancer development, increased carcinogenesis and poor therapy response [86,87,88,89,90,91,92,93,94,95]. 

HPV genotyping of deep sequences relied mainly on querying to NCBI Blastn [72,75]. NCBI Blastn can only query a single sequence per blast search and thus cannot accommodate large sequence data sets. PaVE PV Specific Blastn is able to handle multiple sequences per blast search, but the capacity is far less than 10 Mb per run provided by HPV-QUEST, and genotyping results returned by PaVE PV Specific Blastn require further processing and organization. HPV-QUEST, a web-based genotyping system we developed [33], includes 150 annotated cutaneous and mucosal HPV reference sequences with L1 regions and updated nomenclatures, processes up to 10 Mb of sequences (around 6500 sequences of 100 nt) per run, returns results within one to two minutes, and outputs results in excel and text format, including blast score, e-value, local identity (the percentage of matched nucleotides within the alignment region), genus, species, genotype, infection site (mucosal or cutaneous or both), risk (high or low or unknown), accession number and gene identification number of reference sequence, and alignment of query sequence with reference sequence. Query sequences failing to align with any reference sequences in the HPV-QUEST are designated as “ND”. Any sequences that fail to blast, have low local identity, or with an e-value >1e−15 are considered low quality, a new recombination, or a new genotype. With our NGS/HPV-QUEST genotyping system, large data sets of L1 sequences were easily generated and managed, and were classified rapidly and accurately with stringent quality assurance. The repertoire of HPV genotypes that can be classified unambiguously was extended because all published HPV genotypes were included among the reference sequence database in HPV-QUEST, and analysis was not restricted by availability of HPV type-specific probes. Nevertheless, the consensus nature of PGMY/GP+ primers used in generating amplicon libraries were designed to cover a broad spectrum of HPV types with genetic dissimilarity of at least 10%, rendering amplification bias towards certain HPV types unavoidable, similar to probe-based assays. Less than optimal specificity of GP+ primers for HPV genomes species 3 [96] likely accounts in part for lack of concordance between LA and deep sequencing for HPV typing in some samples. Previous studies suggested that PCR amplification-mediated bias is more due to differential primer-template annealing efficiencies of consensus primers than input template copies [97,98,99].

Sequencing allowed the identification of signature polymorphisms among L1 genotypes in more than 75% of individuals in this exploratory study with relatively small sample size, providing a framework to apply to future studies of HPV clearance, persistence, and super-infection by variants of the same HPV genotypes in a larger cohort. In addition, other regions of the HPV genome that may provide more robust molecular information can be incorporated into a deep sequencing platform to track genotypes over time or evaluate HPV gene expression [100]. A high frequency of nucleotide variants differing from reference variants among a range of HPV genotypes failed to be typed by LA, potentially reflecting an inefficient hybridization between HPV L1 regions with polymorphism and primers and/probes used in LA.

In the current study, deep sequencing was applied to define the range of HPV genotypes in asymptomatic individuals. Our data clearly demonstrate that healthy men can carry a range of high-risk and/or low-risk types HPV genotypes, including the vaccine types. Infection by multiple-type high-risk HPV, alone or in combination with low-risk genotypes, is a significant clinical issue for prognosis or treatment for a variety of HPV cancers [4,13,14,15,16,17,18], while multiple-type infection among asymptomatic individuals can diminish HPV clearance and increase progression to precancerous lesions [8,9,10,11,12,14,19,21,22,23,24,25]. Deep sequencing technology combined with an efficient analysis pipeline offers a platform that can detect HPV genotypes at low frequency in the population, that enables the discovery of the extent to which a repertoire of HPV genotype(s) may relate to subsequent development of disease, that can extend the unambiguous classification of HPV genotypes, and that can provide more robust data about prevalence, dynamics, and competition among genotypes in an ecosystem. Sequence data also provides the basis to implement phylogenetic tools to both assess the organization of HPV genomes within an individual, among transmission pairs, or across geographic locations, and investigate the relationship among new HPV sequences with reference genotypes.

## Supplementary Materials

The following are available online at www.mdpi.com/1999-4915/8/2/28/s1, Table S1: Demographic and sequence profiles of samples, Table S2: HPV genotyping using HPV-QUEST *vs.* PaVE PV Specific Blastn.

## References

[1] D’Souza G., Kreimer A.R., Viscidi R., Pawlita M., Fakhry C., Koch W.M., Westra W.H., Gillison M.L. (2007). Case-control study of human papillomavirus and oropharyngeal cancer. N. Engl. J. Med..

[2] Klein F., Amin Kotb W.F., Petersen I. (2009). Incidence of human papilloma virus in lung cancer. Lung Cancer.

[3] Li N., Yang L., Zhang Y., Zhao P., Zheng T., Dai M. (2011). Human papillomavirus infection and bladder cancer risk: A meta-analysis. J. Infect. Dis..

[4] Marur S., D’Souza G., Westra W.H., Forastiere A.A. (2010). HPV-associated head and neck cancer: A virus-related cancer epidemic. Lancet Oncol..

[5] Walboomers J.M., Jacobs M.V., Manos M.M., Bosch F.X., Kummer J.A., Shah K.V., Snijders P.J., Peto J., Meijer C.J., Munoz N. (1999). Human papillomavirus is a necessary cause of invasive cervical cancer worldwide. J. Pathol..

[6] Gallegos-Hernandez J.F., Paredes-Hernandez E., Flores-Diaz R., Minauro-Munoz G., Apresa-Garcia T., Hernandez-Hernandez D.M. (2007). Human papillomavirus: Association with head and neck cancer. Cir. Cir..

[7] Giuliano A.R., Nyitray A.G., Kreimer A.R., Pierce Campbell C.M., Goodman M.T., Sudenga S.L., Monsonego J., Franceschi S. (2015). EUROGIN 2014 roadmap: Differences in human papillomavirus infection natural history, transmission and human papillomavirus-related cancer incidence by gender and anatomic site of infection. Int. J. Cancer.

[8] Anwar K., Naiki H., Nakakuki K., Inuzuka M. (1992). High frequency of human papillomavirus infection in carcinoma of the urinary bladder. Cancer.

[9] Fife K.H., Cramer H.M., Schroeder J.M., Brown D.R. (2001). Detection of multiple human papillomavirus types in the lower genital tract correlates with cervical dysplasia. J. Med. Virol..

[10] Sasagawa T., Basha W., Yamazaki H., Inoue M. (2001). High-risk and multiple human papillomavirus infections associated with cervical abnormalities in Japanese women. Cancer Epidemiol. Biomark. Prev..

[11] Yang Y., Li X., Zhang Z., Qian H.Z., Ruan Y., Zhou F., Gao C., Li M., Jin Q., Gao L. (2012). Association of human papillomavirus infection and abnormal anal cytology among HIV-infected MSM in Beijing, China. PLoS ONE.

[12] Trottier H., Mahmud S., Costa M.C., Sobrinho J.P., Duarte-Franco E., Rohan T.E., Ferenczy A., Villa L.L., Franco E.L. (2006). Human papillomavirus infections with multiple types and risk of cervical neoplasia. Cancer Epidemiol. Biomark. Prev..

[13] Bachtiary B., Obermair A., Dreier B., Birner P., Breitenecker G., Knocke T.H., Selzer E., Potter R. (2002). Impact of multiple HPV infection on response to treatment and survival in patients receiving radical radiotherapy for cervical cancer. Int. J. Cancer.

[14] Munagala R., Dona M.G., Rai S.N., Jenson A.B., Bala N., Ghim S.J., Gupta R.C. (2009). Significance of multiple HPV infection in cervical cancer patients and its impact on treatment response. Int. J. Oncol..

[15] Syrjanen S. (2010). The role of human papillomavirus infection in head and neck cancers. Ann. Oncol..

[16] Dayyani F., Etzel C.J., Liu M., Ho C.H., Lippman S.M., Tsao A.S. (2010). Meta-analysis of the impact of human papillomavirus (HPV) on cancer risk and overall survival in head and neck squamous cell carcinomas (HNSCC). Head Neck Oncol..

[17] Ragin C.C., Taioli E. (2007). Survival of squamous cell carcinoma of the head and neck in relation to human papillomavirus infection: Review and meta-analysis. Int. J. Cancer.

[18] Rautava J., Kuuskoski J., Syrjanen K., Grenman R., Syrjanen S. (2012). HPV genotypes and their prognostic significance in head and neck squamous cell carcinomas. J. Clin. Virol..

[19] Nielson C.M., Harris R.B., Flores R., Abrahamsen M., Papenfuss M.R., Dunne E.F., Markowitz L.E., Giuliano A.R. (2009). Multiple-type human papillomavirus infection in male anogenital sites: Prevalence and associated factors. Cancer Epidemiol. Biomark. Prev..

[20] Kjaer S.K., Munk C., Winther J.F., Jorgensen H.O., Meijer C.J., van den Brule A.J. (2005). Acquisition and persistence of human papillomavirus infection in younger men: A prospective follow-up study among Danish soldiers. Cancer Epidemiol. Biomark. Prev..

[21] Louvanto K., Syrjanen K.J., Rintala M.A., Grenman S.E., Syrjanen S.M. (2010). Genotype-specific clearance of genital human papillomavirus (HPV) infections among mothers in the Finnish family HPV study. J. Clin. Microbiol..

[22] Louvanto K., Rintala M.A., Syrjanen K.J., Grenman S.E., Syrjanen S.M. (2010). Genotype-specific persistence of genital human papillomavirus (HPV) infections in women followed for 6 years in the Finnish Family HPV Study. J. Infect. Dis..

[23] Kovacs K., Varnai A.D., Bollmann M., Bankfalvi A., Szendy M., Speich N., Schmitt C., Pajor L., Bollmann R., Hildenbrand R. (2009). A 7.5-year prospective study of longer than 18 months type-specific human papillomavirus persistence in a routine cytology-based cervical screening population of about 31,000 women in West Germany. Eur. J. Cancer Prev..

[24] Chua K.L., Hjerpe A. (1996). Persistence of human papillomavirus (HPV) infections preceding cervical carcinoma. Cancer.

[25] Yang Z., Cuzick J., Hunt W.C., Wheeler C.M. (2014). Concurrence of multiple human papillomavirus infections in a large US population-based cohort. Am. J. Epidemiol..

[26] Carvalho N.O., Del Castillo D.M., Perone C., Januario J.N., Melo V.H., Brasileiro F.G. (2010). Comparison of HPV genotyping by type-specific PCR and sequencing. Mem. Inst. Oswaldo Cruz.

[27] Castle P.E., Porras C., Quint W.G., Rodriguez A.C., Schiffman M., Gravitt P.E., Gonzalez P., Katki H.A., Silva S., Freer E. (2008). Comparison of two PCR-based human papillomavirus genotyping methods. J. Clin. Microbiol..

[28] Klug S.J., Molijn A., Schopp B., Holz B., Iftner A., Quint W., Snijders J.F., Petry K.U., Kruger K.S., Munk C. (2008). Comparison of the performance of different HPV genotyping methods for detecting genital HPV types. J. Med. Virol..

[29] Plummer M., Vaccarella S., Franceschi S. (2011). Multiple human papillomavirus infections: The exception or the rule?. J. Infect. Dis..

[30] Wang C.C., Lai C.H., Huang H.J., Chao A., Chang C.J., Chang T.C., Chou H.H., Hong J.H. (2010). Clinical effect of human papillomavirus genotypes in patients with cervical cancer undergoing primary radiotherapy. Int. J. Radiat. Oncol. Biol. Phys..

[31] Gharizadeh B., Kalantari M., Garcia C.A., Johansson B., Nyren P. (2001). Typing of human papillomavirus by pyrosequencing. Lab. Investig..

[32] Gharizadeh B., Oggionni M., Zheng B., Akom E., Pourmand N., Ahmadian A., Wallin K.L., Nyren P. (2005). Type-specific multiple sequencing primers: A novel strategy for reliable and rapid genotyping of human papillomaviruses by pyrosequencing technology. J. Mol. Diagn..

[33] Yin L., Yao J., Gardner B.P., Chang K., Yu F., Goodenow M.M. (2012). HPV-QUEST: A highly customized system for automated HPV sequence analysis capable of processing Next Generation sequencing data set. Bioinformation.

[34] Giuliano A.R., Lazcano-Ponce E., Villa L.L., Flores R., Salmeron J., Lee J.H., Papenfuss M.R., Abrahamsen M., Jolles E., Nielson C.M. (2008). The human papillomavirus infection in men study: Human papillomavirus prevalence and type distribution among men residing in Brazil, Mexico, and the United States. Cancer Epidemiol. Biomark. Prev..

[35] Giuliano A.R., Lee J.H., Fulp W., Villa L.L., Lazcano E., Papenfuss M.R., Abrahamsen M., Salmeron J., Anic G.M., Rollison D.E. (2011). Incidence and clearance of genital human papillomavirus infection in men (HIM): A cohort study. Lancet.

[36] Lu B., Viscidi R.P., Lee J.H., Wu Y., Villa L.L., Lazcano-Ponce E., da Silva R.J., Baggio M.L., Quiterio M., Salmeron J. (2011). Human papillomavirus (HPV) 6, 11, 16, and 18 seroprevalence is associated with sexual practice and age: Results from the multinational HPV Infection in Men Study (HIM Study). Cancer Epidemiol. Biomark. Prev..

[37] Vaccarella S., Plummer M., Franceschi S., Gravitt P., Papenfuss M., Smith D., Villa L., Ponce E.L., Giuliano A.R. (2011). Clustering of human papillomavirus (HPV) types in the male genital tract: The HPV in men (HIM) study. J. Infect. Dis..

[38] Flores R., Abalos A.T., Nielson C.M., Abrahamsen M., Harris R.B., Giuliano A.R. (2008). Reliability of sample collection and laboratory testing for HPV detection in men. J. Virol. Methods.

[39] Benki S., McClelland R.S., Emery S., Baeten J.M., Richardson B.A., Lavreys L., Mandaliya K., Overbaugh J. (2006). Quantification of genital human immunodeficiency virus type 1 (HIV-1) DNA in specimens from women with low plasma HIV-1 RNA levels typical of HIV-1 nontransmitters. J. Clin. Microbiol..

[40] Kumar R., Vandegraaff N., Mundy L., Burrell C.J., Li P. (2002). Evaluation of PCR-based methods for the quantitation of integrated HIV-1 DNA. J. Virol. Methods.

[41] Montoya J.G., Wood R., Katzenstein D., Holodny M., Merigan T.C. (1993). Peripheral blood mononuclear cell human immunodeficiency virus type 1 proviral DNA quantification by polymerase chain reaction: Relationship to immunodeficiency and drug effect. J. Clin. Microbiol..

[42] Vandegraaff N., Kumar R., Burrell C.J., Li P. (2001). Kinetics of human immunodeficiency virus type 1 (HIV) DNA integration in acutely infected cells as determined using a novel assay for detection of integrated HIV DNA. J. Virol..

[43] Yee C., Krishnan-Hewlett I., Baker C.C., Schlegel R., Howley P.M. (1985). Presence and expression of human papillomavirus sequences in human cervical carcinoma cell lines. Am. J. Pathol..

[44] Gravitt P.E., Peyton C.L., Alessi T.Q., Wheeler C.M., Coutlee F., Hildesheim A., Schiffman M.H., Scott D.R., Apple R.J. (2000). Improved amplification of genital human papillomaviruses. J. Clin. Microbiol..

[45] De Roda Husman A.M., Walboomers J.M., van den Brule A.J., Meijer C.J., Snijders P.J. (1995). The use of general primers GP5 and GP6 elongated at their 3′ ends with adjacent highly conserved sequences improves human papillomavirus detection by PCR. J. Gen. Virol..

[46] Fuessel Haws A.L., He Q., Rady P.L., Zhang L., Grady J., Hughes T.K., Stisser K., Konig R., Tyring S.K. (2004). Nested PCR with the PGMY09/11 and GP5(+)/6(+) primer sets improves detection of HPV DNA in cervical samples. J. Virol. Methods.

[47] Winder D.M., Ball S.L., Vaughan K., Hanna N., Woo Y.L., Franzer J.T., Sterling J.C., Stanley M.A., Sudhoff H., Goon P.K. (2009). Sensitive HPV detection in oropharyngeal cancers. BMC Cancer.

[48] Jacobs M.V., Snijders P.J., Voorhorst F.J., Dillner J., Forslund O., Johansson B., von Knebel D.M., Meijer C.J., Meyer T., Nindl I. (1999). Reliable high risk HPV DNA testing by polymerase chain reaction: An intermethod and intramethod comparison. J. Clin. Pathol..

[49] Kornegay J.R., Roger M., Davies P.O., Shepard A.P., Guerrero N.A., Lloveras B., Evans D., Coutlee F. (2003). International proficiency study of a consensus L1 PCR assay for the detection and typing of human papillomavirus DNA: Evaluation of accuracy and intralaboratory and interlaboratory agreement. J. Clin. Microbiol..

[50] (2009). Roche: Amplicon Fusion Primer Design Guidlines for GS FLX Titanium Seris Lib-A Chemistry.

[51] Sun Y., Cai Y., Liu L., Yu F., Farrell M.L., McKendree W., Farmerie W. (2009). ESPRIT: Estimating species richness using large collections of 16S rRNA pyrosequences. Nucleic Acids Res..

[52] Bernard H.U., Calleja-Macias I.E., Dunn S.T. (2006). Genome variation of human papillomavirus types: Phylogenetic and medical implications. Int. J. Cancer.

[53] De Villiers E.M., Fauquet C., Broker T.R., Bernard H.U., Zur H.H. (2004). Classification of papillomaviruses. Virology.

[54] Yamada T., Wheeler C.M., Halpern A.L., Stewart A.C., Hildesheim A., Jenison S.A. (1995). Human papillomavirus type 16 variant lineages in United States populations characterized by nucleotide sequence analysis of the E6, L2, and L1 coding segments. J. Virol..

[55] NCBI Genebank. http://www.ncbi.nlm.nih.gov/Taxonomy/Browser/wwwtax.cgi?mode=Tree&id=151340&lvl=3&lin=f&keep=1&srchmode=1&unlock.

[56] Los Alamos HPV Sequence Database. http://hpv-web.lanl.gov/.

[57] Virus Sequence Database. http://kcdc.labkm.net/vsd/database/gene_search_7.jsp?orgId=7&reset=1.

[58] Chan S.Y., Delius H., Halpern A.L., Bernard H.U. (1995). Analysis of genomic sequences of 95 papillomavirus types: Uniting typing, phylogeny, and taxonomy. J. Virol..

[59] Papillomavirus Episteme Blastn. http://pave.niaid.nih.gov/#search/pv_specific_blast.

[60] NCBI Blastn. http://blast.ncbi.nlm.nih.gov/Blast.cgi?PROGRAM=blastn&PAGE_TYPE=BlastSearch&LINK_LOC=blasthome.

[61] Bernard H.U., Chan S.Y., Manos M.M., Ong C.K., Villa L.L., Delius H., Peyton C.L., Bauer H.M., Wheeler C.M. (1994). Identification and assessment of known and novel human papillomaviruses by polymerase chain reaction amplification, restriction fragment length polymorphisms, nucleotide sequence, and phylogenetic algorithms. J. Infect. Dis..

[62] Delius H., Hofmann B. (1994). Primer-directed sequencing of human papillomavirus types. Curr. Top. Microbiol. Immunol..

[63] Schwarz E., Durst M., Demankowski C., Lattermann O., Zech R., Wolfsperger E., Suhai S., zur Hausen H. (1983). DNA sequence and genome organization of genital human papillomavirus type 6b. EMBO J..

[64] Chaturvedi A.K., Katki H.A., Hildesheim A., Rodriguez A.C., Quint W., Schiffman M., van Doorn L.J., Porras C., Wacholder S., Gonzalez P. (2011). Human papillomavirus infection with multiple types: Pattern of coinfection and risk of cervical disease. J. Infect. Dis..

[65] Westra W.H. (2014). Detection of human papillomavirus (HPV) in clinical samples: Evolving methods and strategies for the accurate determination of HPV status of head and neck carcinomas. Oral Oncol..

[66] Mirghani H., Amen F., Moreau F., Guigay J., Ferchiou M., Melkane A.E., Hartl D.M., Lacau St G.J. (2014). Human papilloma virus testing in oropharyngeal squamous cell carcinoma: What the clinician should know. Oral Oncol..

[67] Robinson M., Sloan P., Shaw R. (2010). Refining the diagnosis of oropharyngeal squamous cell carcinoma using human papillomavirus testing. Oral Oncol..

[68] Kleter B., van Doorn L.J., ter Schegget J., Schrauwen L., van K.K., Burger M., ter Harmsel B., Quint W. (1998). Novel short-fragment PCR assay for highly sensitive broad-spectrum detection of anogenital human papillomaviruses. Am. J. Pathol..

[69] Kleter B., van Doorn L.J., Schrauwen L., Molijn A., Sastrowijoto S., ter Schegget J., Lindeman J., ter Harmsel B., Burger M., Quint W. (1999). Development and clinical evaluation of a highly sensitive PCR-reverse hybridization line probe assay for detection and identification of anogenital human papillomavirus. J. Clin. Microbiol..

[70] Hwang T.S., Jeong J.K., Park M., Han H.S., Choi H.K., Park T.S. (2003). Detection and typing of HPV genotypes in various cervical lesions by HPV oligonucleotide microarray. Gynecol. Oncol..

[71] van den Brule A.J., Pol R., Fransen-Daalmeijer N., Schouls L.M., Meijer C.J., Snijders P.J. (2002). GP5+/6+ PCR followed by reverse line blot analysis enables rapid and high-throughput identification of human papillomavirus genotypes. J. Clin. Microbiol..

[72] Arroyo L.S., Smelov V., Bzhalava D., Eklund C., Hultin E., Dillner J. (2013). Next generation sequencing for human papillomavirus genotyping. J. Clin. Virol..

[73] Vernon S.D., Unger E.R., Williams D. (2000). Comparison of human papillomavirus detection and typing by cycle sequencing, line blotting, and hybrid capture. J. Clin. Microbiol..

[74] Barzon L., Lavezzo E., Militello V., Toppo S., Palu G. (2011). Applications of next-generation sequencing technologies to diagnostic virology. Int. J. Mol. Sci..

[75] Meiring T.L., Salimo A.T., Coetzee B., Maree H.J., Moodley J., Hitzeroth I.I., Freeborough M.J., Rybicki E.P., Williamson A.L. (2012). Next-generation sequencing of cervical DNA detects human papillomavirus types not detected by commercial kits. Virol. J..

[76] Militello V., Lavezzo E., Costanzi G., Franchin E., Di C.B., Toppo S., Palu G., Barzon L. (2013). Accurate human papillomavirus genotyping by 454 pyrosequencing. Clin. Microbiol. Infect..

[77] Ekstrom J., Bzhalava D., Svenback D., Forslund O., Dillner J. (2011). High throughput sequencing reveals diversity of Human Papillomaviruses in cutaneous lesions. Int. J. Cancer.

[78] Johansson H., Bzhalava D., Ekstrom J., Hultin E., Dillner J., Forslund O. (2013). Metagenomic sequencing of “HPV-negative” condylomas detects novel putative HPV types. Virology.

[79] Ho L., Chan S.Y., Burk R.D., Das B.C., Fujinaga K., Icenogle J.P., Kahn T., Kiviat N., Lancaster W., Mavromara-Nazos P. (1993). The genetic drift of human papillomavirus type 16 is a means of reconstructing prehistoric viral spread and the movement of ancient human populations. J. Virol..

[80] Lichtig H., Algrisi M., Botzer L.E., Abadi T., Verbitzky Y., Jackman A., Tommasino M., Zehbe I., Sherman L. (2006). HPV16 E6 natural variants exhibit different activities in functional assays relevant to the carcinogenic potential of E6. Virology.

[81] Ong C.K., Chan S.Y., Campo M.S., Fujinaga K., Mavromara-Nazos P., Labropoulou V., Pfister H., Tay S.K., ter Meulen J., Villa L.L. (1993). Evolution of human papillomavirus type 18: An ancient phylogenetic root in Africa and intratype diversity reflect coevolution with human ethnic groups. J. Virol..

[82] Pillai M.R., Hariharan R., Babu J.M., Lakshmi S., Chiplunkar S.V., Patkar M., Tongaonkar H., Dinshaw K., Jayshree R.S., Reddy B.K. (2009). Molecular variants of HPV-16 associated with cervical cancer in Indian population. Int. J. Cancer.

[83] Travasso C.M., Anand M., Samarth M., Deshpande A., Kumar-Sinha C. (2008). Human papillomavirus genotyping by multiplex pyrosequencing in cervical cancer patients from India. J. Biosci..

[84] Chan P.K., Cheung T.H., Tam A.O., Lo K.W., Yim S.F., Yu M.M., To K.F., Wong Y.F., Cheung J.L., Chan D.P. (2006). Biases in human papillomavirus genotype prevalence assessment associated with commonly used consensus primers. Int. J. Cancer.

[85] Eklund C., Forslund O., Wallin K.L., Zhou T., Dillner J. (2012). The 2010 global proficiency study of human papillomavirus genotyping in vaccinology. J. Clin. Microbiol..

[86] Al-Shabanah O.A., Hafez M.M., Hassan Z.K., Sayed-Ahmed M.M., Abozeed W.N., Al-Rejaie S.S., Alsheikh A.A. (2013). Human papillomavirus genotyping and integration in ovarian cancer Saudi patients. Virol. J..

[87] Briolat J., Dalstein V., Saunier M., Joseph K., Caudroy S., Pretet J.L., Birembaut P., Clavel C. (2007). HPV prevalence, viral load and physical state of HPV-16 in cervical smears of patients with different grades of CIN. Int. J. Cancer.

[88] Cricca M., Morselli-Labate A.M., Venturoli S., Ambretti S., Gentilomi G.A., Gallinella G., Costa S., Musiani M., Zerbini M. (2007). Viral DNA load, physical status and E2/E6 ratio as markers to grade HPV16 positive women for high-grade cervical lesions. Gynecol. Oncol..

[89] Ho C.M., Lee B.H., Chang S.F., Chien T.Y., Huang S.H., Yan C.C., Cheng W.F. (2011). Integration of human papillomavirus correlates with high levels of viral oncogene transcripts in cervical carcinogenesis. Virus Res..

[90] Melsheimer P., Vinokurova S., Wentzensen N., Bastert G., von Knebel D.M. (2004). DNA aneuploidy and integration of human papillomavirus type 16 e6/e7 oncogenes in intraepithelial neoplasia and invasive squamous cell carcinoma of the cervix uteri. Clin. Cancer Res..

[91] Pett M., Coleman N. (2007). Integration of high-risk human papillomavirus: A key event in cervical carcinogenesis?. J. Pathol..

[92] Shin H.J., Joo J., Yoon J.H., Yoo C.W., Kim J.Y. (2014). Physical status of human papillomavirus integration in cervical cancer is associated with treatment outcome of the patients treated with radiotherapy. PLoS ONE.

[93] Wentzensen N., Vinokurova S., von Knebel D.M. (2004). Systematic review of genomic integration sites of human papillomavirus genomes in epithelial dysplasia and invasive cancer of the female lower genital tract. Cancer Res..

[94] Xu B., Chotewutmontri S., Wolf S., Klos U., Schmitz M., Durst M., Schwarz E. (2013). Multiplex identification of human papillomavirus 16 DNA integration sites in cervical carcinomas. PLoS ONE.

[95] Ziegert C., Wentzensen N., Vinokurova S., Kisseljov F., Einenkel J., Hoeckel M., von Knebel D.M. (2003). A comprehensive analysis of HPV integration loci in anogenital lesions combining transcript and genome-based amplification techniques. Oncogene.

[96] Speich N., Schmitt C., Bollmann R., Bollmann M. (2004). Human papillomavirus (HPV) study of 2916 cytological samples by PCR and DNA sequencing: Genotype spectrum of patients from the west German area. J. Med. Microbiol..

[97] Polz M.F., Cavanaugh C.M. (1998). Bias in template-to-product ratios in multitemplate PCR. Appl. Environ. Microbiol..

[98] Qu W., Jiang G., Cruz Y., Chang C.J., Ho G.Y., Klein R.S., Burk R.D. (1997). PCR detection of human papillomavirus: Comparison between MY09/MY11 and GP5+/GP6+ primer systems. J. Clin. Microbiol..

[99] Suzuki M.T., Giovannoni S.J. (1996). Bias caused by template annealing in the amplification of mixtures of 16S rRNA genes by PCR. Appl. Environ. Microbiol..

[100] Lei Y.J., Makhaola K., Pittayakhajonwut D., Wood C., Angeletti P.C. (2011). Human papillomavirus 16 variants from Zambian women with normal pap smears. J. Med. Virol..

