# Supplementary material for: HPV Population Profiling in Healthy Men by Next-Generation Deep Sequencing Coupled with HPV-QUEST
Source: Viruses. 2016 Jan 25;8(2):28. doi: 10.3390/v8020028 (PMC4776183; doi:10.3390/v8020028)
Supplement: Supplementary file 1 [file viruses-08-00028-s001.pdf]

Supplementary Materials: HPV Population Profiling in Healthy Men by Next Generation Deep Sequencing Coupled with HPV-QUEST

Li Yin, Jin Yao, Kaifen Chang, Brent P. Gardner, Fahong Yu, Anna R. Giuliano and Maureen M. Goodenow

Table S1. Demographic and sequence profiles of samples.

Sample ID	HPV Genotype(s) Identified by Deep Sequencing Number [genotype]	Sex Partners # (number)	Age (year)	Country of Origin	Sequence Number		
					Raw Reads ‡	Mapped Quality Sequences	
						Individual Sequences	Consensus Sequences
**S1**	1 [6b]	0	19	US	1806	1550	8
**S2**	1 [6b]	1	22	US	685	633	2
**S3**	1 [16]	N/A ¶	19	US	1468	1362	6
**S4**	1 [16]	0	20	US	2061	1891	7
**S5**	1 [16]	3	20	US	145	121	1
**S6**	1 [83]	3	27	US	1656	1519	2
**S7**	3 [6b/83/87]	1	23	US	1344	1015	1/1/1
**S8**	3 [16/18/6b]	1	20	US	828	701	1/2/1
**S9**	3 [16/52/59]	3	19	US	1686	1534	1/1/6
**S10**	2 [70/73]	3	21	US	2128	1866	1/5
**S11**	3 [73/cand85/6b]	0	19	US	1899	1698	1/1/2
**S12**	4 [16/35/66/6b]	0	32	US	1808	1660	1/1/1/1
**S13**	5 [70/73/cand85/32/JEB2]	0	52	US	456	409	1/1/1/1/3
**S14**	5 [16/31/56/66/62]	0	31	US	991	941	2/1/1/1/1
**S15 (T0)**	2 [16/51]	1	22	US	50,061	49,060	1/1
**S15 (T1)**	2 [16/81]	2	22		16,393	15,702	1/1
**S15 (T2)**	2 [16/83]	1	23		18,808	18,161	1/1
**S16 (T0)**	2 [16/11]	0	36	Mexico	13,183	12,584	1/1
**S16 (T1)**	1 [16]	0	37		27,376	26,988	1
**S16 (T2)**	1 [16]	0	38		31,440	31,236	1
**S17**	1 [16]	0	41	Mexico	36,771	36,038	1
**S18**	2 [81/cand89]	1	43	Brazil	43,698	43,599	3/1
**C-4 II**	1	-	-	-	16,586	16,444	1
**CaSki**	1	-	-	-	22,924	22,763	1
**HPV16 ***	1	-	-	-	26,193	25,928	1

*: a molecular clone; #: number of sex partner(s) within 6 weeks prior to sampling; ¶: information unavailable; ‡: >50% of base calls within each read with Phred quality score ≥ 40.

Table S2. HPV genotyping using HPV-QUEST vs. PaVE PV Specific Blastn.

Sample ID	HPV-QUEST					PaVE PV Specific Blastn				
	Type (Variant)	GI	Score	E Value	Local Identity	Type (Variant)	GI	Score	E Value	Local Identity
S1	6b (1)	60955	180	4E-47	91/91	6b (1)	60955	180	7E-47	91/91
	6b (2)	60955	170	4E-44	89/90	6b (2)	60955	170	6E-44	89/90
	6b (3)	60955	162	9E-42	88/90	6b (3)	60955	163	2E-41	88/90
	6b (4)	60955	170	4E-44	89/90	6b (4)	60955	170	6E-44	89/90
	6b (5)	60955	162	9E-42	88/90	6b (5)	60955	163	2E-41	88/90
	6b (6)	60955	162	9E-42	88/90	6b (6)	60955	163	2E-41	88/90
	6b (7)	60955	168	1E-43	88/89	6b (7)	60955	168	2E-43	88/89
	6b (8)	60955	166	6E-43	87/88	6b (8)	60955	167	1E-42	87/88
S2	6b (1)	60955	180	4E-47	91/91	6b (1)	60955	180	7E-47	91/91
	6b (2)	60955	162	9E-42	88/90	6b (2)	60955	163	2E-41	89/90
S3	16 (1)	333031	186	7E-49	94/94	16 (1)	333031	186	1E-48	94/94
	16 (2)	333031	168	2E-43	91/93	16 (2)	333031	168	3E-43	91/93
	16 (3)	333031	168	2E-43	91/93	16 (3)	333031	168	3E-43	91/93
	16 (4)	333031	168	2E-43	91/93	16 (4)	333031	168	3E-43	91/93
	16 (5)	333031	168	2E-43	91/93	16 (5)	333031	168	3E-43	91/93
	16 (6)	333031	168	2E-43	91/93	16 (6)	333031	168	3E-43	91/93
S4	16 (1)	333031	186	7E-49	94/94	16 (1)	333031	186	1E-48	94/94
	16 (2)	333031	168	2E-43	91/93	16 (2)	333031	168	3E-43	91/93
	16 (3)	333031	160	4E-41	90/93	16 (3)	333031	161	6E-41	90/93
	16 (4)	333031	176	6E-46	92/93	16 (4)	333031	176	1E-45	92/93
	16 (5)	333031	170	4E-44	92/94	16 (5)	333031	170	7E-44	92/94
	16 (6)	333031	168	2E-43	91/93	16 (6)	333031	163	2E-41	91/93
	16 (7)	333031	168	2E-43	91/93	16 (7)	333031	168	3E-43	91/93
S5	16	333031	186	7E-49	94/94	16	333031	186	1E-48	94/94
S6	83 (1)	5059324	172	1E-44	87/87	83 (1)	5059324	172	2E-44	87/87
	83 (2)	5059324	160	4E-41	84/85	83 (2)	5059324	161	6E-41	84/85
S7	6b	60955	178	2E-46	90/90	6b	60955	178	3E-46	90/90
	83	5059324	172	1E-44	87/87	83	5059324	172	2E-44	87/87
	87	14475578	172	9E-45	87/87	87	14475578	172	2E-44	87/87
S8	16	333031	176	6E-46	92/93	16	333031	176	1E-45	92/93
	18 (1)	60975	186	7E-49	94/94	18 (1)	60975	192	2E-50	97/97
	18 (2)	60975	174	3E-45	91/92	18 (2)	60975	178	3E-46	93/94
	6b	60955	239	9E-65	130/133	6b	60955	240	1E-64	130/133
S9	16	333031	186	7E-49	94/94	16	333031	186	1E-48	94/94
	52	397038	180	4E-47	91/91	52	397038	180	7E-47	91/91
	59 (1)	557236	192	1E-50	97/97	59 (1)	557236	192	2E-50	97/97
	59 (2)	557236	174	3E-45	94/96	59 (2)	557236	174	4E-45	94/96
	59 (3)	557236	166	6E-43	93/96	59 (3)	557236	167	1E-42	93/96
	59 (4)	557236	174	3E-45	91/92	59 (4)	557236	174	4E-45	91/92
	59 (5)	557236	174	3E-45	94/96	59 (5)	557236	174	4E-45	94/96
	59 (6)	557236	174	3E-45	94/96	59 (6)	557236	174	4E-45	94/96
S10	70	1173493	188	2E-49	98/99	70	1173493	188	3E-49	98/99
	73 (1)	1491692	190	5E-50	99/100	73 (1)	1491692	190	8E-50	99/100
	73 (2)	1491692	166	7E-43	93/96	73 (2)	1491692	167	1E-42	93/96
	73 (3)	1491692	166	7E-43	93/96	73 (3)	1491692	167	1E-42	93/96
	73 (4)	1491692	166	7E-43	96/100	73 (4)	1491692	167	1E-42	96/100
	73 (5)	1491692	166	7E-43	96/100	73 (5)	1491692	167	1E-42	96/100
S11	73	1491692	156	5E-40	85/87	73	1491692	157	9E-40	85/87
	Cand85	4574720	174	3E-45	94/96	Cand85	4574720	174	4E-45	94/96
	6b (1)	60955	180	4E-47	91/91	6b (1)	60955	180	7E-47	91/91
	6b (2)	60955	152	9E-39	86/89	6b (2)	60955	157	9E-40	85/87
S12	16	333031	186	7E-49	94/94	16	333031	186	1E-48	94/94
	35h	396997	178	2E-46	93/94	35h	396997	178	3E-46	93/94
	66	1020290	180	4E-47	91/91	66	1020290	180	7E-47	91/91
	6b	60955	178	2E-46	90/90	6b	60955	178	3E-46	90/90

Table S2. *Cont.*

Sample ID	HPV-QUEST					PaVE PV Specific Blastn				
	Type (Variant)	GI	Score	E Value	Local Identity	Type (Variant)	GI	Score	E Value	Local Identity
**S13**	70	1173493	184	3E-48	96/97	70	1173493	184	5E-48	96/97
	73	1491692	152	7E-39	77/77	73	1491692	153	1E-38	77/77
	Cand85	4574720	184	3E-48	93/93	Cand85	4574720	184	4E-48	93/93
	**32 ***	**396981**	180	4E-47	91/91	**32**	**9627327**	180	7E-47	91/91
	**JEB2 (1) #**	**45925861**	162	9E-42	82/82	**72 (1)**	**1491683**	100	2E-22	80/90
	**JEB2 (2) #**	**45925861**	146	5E-37	77/78	**72 (2)**	**1491683**	84	1E-17	75/86
	**JEB2 (3) #**	**45925861**	146	5E-37	80/82	**72 (3)**	**1491683**	84	1E-17	78/90
**S14**	16 (1)	333031	186	7E-49	94/94	16 (1)	333031	186	1E-48	94/94
	16 (2)	333031	170	4E-44	89/90	16 (2)	333031	170	7E-44	89/90
	31	333048	170	4E-44	92/94	31	333048	170	7E-44	92/94
	56	39053	180	4E-47	91/91	56	39053	180	7E-47	91/91
	66	1020290	186	7E-49	94/94	66	1020290	186	1E-48	94/94
	**62 §**	**577400**	172	1E-44	90/91	**62**	**39932599**	172	2E-44	87/87
**S15(T0)**	16	333031	182	1E-47	92/92	16	333031	182	2E-47	92/92
	51	333087	180	1E-46	91/91	51]	333087	180	7E-47	91/91
**S15(T1)**	16	333031	180	4E-47	91/91	16	333031	180	7E-47	91/91
	81	40804509	172	1E-44	90/91	81	40804509	172	2E-44	90/91
**S15(T2)**	16	333031	180	4E-47	91/91	16	333031	180	7E-47	91/91
	83	5059324	164	2E-42	83/83	83	5059324	165	4E-42	83/83
**S16(T0)**	16	333031	182	1E-47	92/92	16	333031	182	2E-47	92/92
	11	333026	170	9E-44	90/91	11	333026	165	4E-42	90/91
**S16(T1)**	16	333031	182	1E-47	92/92	16	333031	182	2E-47	92/92
**S16(T2)**	16	333031	182	1E-47	92/92	16	333031	182	2E-47	92/92
**S17**	16	333031	231	2E-62	117/117	16	333031	232	3E-62	117/117
**S18**	81 (1)	40804509	178	2E-46	90/90	81 (1)	40804509	178	3E-46	90/90
	81 (2)	40804509	170	4E-44	89/90	81 (2)	40804509	170	6E-44	89/90
	81 (3)	40804509	162	9E-42	88/90	81 (3)	40804509	163	2E-41	88/90
	Cand89	22095322	164	2E-42	90/92	Cand89	22095322	159	2E-40	90/92
**C-4 II**	18	60975	186	7E-49	94/94	18	60975	192	2E-50	97/97
**CaSki**	16	333031	186	7E-49	94/94	16	333031	186	1E-48	94/94
**HPV16clone**	16	333031	186	7E-49	94/94	16	333031	186	1E-48	94/94

Genotyping by NCBI Blastn: *, HPV32 (GI│ 507143814); #, HPVJEB2 (GI│ 217885000); §, HPV62 (GI│ 577400).

© 2016 by the authors; licensee MDPI, Basel, Switzerland. This article is an open access article distributed under the terms and conditions of the Creative Commons by Attribution (CC-BY) license (http://creativecommons.org/licenses/by/4.0/).
